# The Correlation of Pirani and Dimeglio Scoring Systems for Ponseti Management at Different Levels of Deformity Severity

**DOI:** 10.1038/s41598-017-14977-7

**Published:** 2017-11-06

**Authors:** Hua Fan, Yubin Liu, Li Zhao, Caiting Chu, Yongyu An, Tingting Wang, Wenhua Li

**Affiliations:** 1 Department of Radiology, Xinhua Hospital, Shanghai Jiaotong University School of Medicine, Shanghai, 200092 China; 2 Department of Pediatric Orthopaedics Xinhua Hospital, Shanghai Jiaotong University School of Medicine, Shanghai, 200092 China; 3 Department of Orthopaedics, Zhujiang Hospital of Southern Medical University, Guangzhou, 510282 China

## Abstract

The Pirani and Dimeglio scoring systems both have excellent inter-observer and intra-observer reliability, but no research has been conducted to determine their inter-observer reliability and their relationship at different levels of deformity. A total of 173 idiopathic clubfoot cases were reviewed using Pirani and Dimeglio scoring systems, and the number of casts needed was also recorded. For clubfeet with a cast number equal to 2 or 7 and 8, the inter-observer reliability of the two scoring systems was poor or moderate, and there was no correlation between the two scoring systems. There was also no correlation between the Dimeglio scoring score with the number of casts for grade II or IV clubfeet. A binary regression of the number of casts on initial Pirani or Dimeglio scores showed that there was a Quadratic or Cubic relation between the scores and the cast numbers. In conclusion, in the case of mild and very severe clubfoot deformity, the interobserver reliability and its ability to predict the number of casts needed for clubfoot deformity correction was poor. A more objective evaluation system may be required.

## Introduction

Clubfoot is one of the most prevalent musculoskeletal congenital defects, with an incidence from 0.9 to 7 of 1000 live births^1^. The deformity is not self-healing, and if timely treatment does not occur (the age at the onset of treatment need further study^2–5^), the deformity will deteriorate until adulthood and cause adverse effects for the patient. Because its aetiology is not thoroughly understood, we cannot provide aetiological treatment. The aim of the treatment for clubfoot is to achieve a functionally sound, painless, and cosmetically acceptable foot for the patient. For decades, surgeons have been pursuing surgical methods to rectify the deformity to the normal anatomy of the foot, but long-term follow-up results have shown that the surgeries left many patients with dynamic stiffness and pain^6^. Current treatment of clubfoot has moved away from operative treatment to conservative treatment^7^. The Ponseti method has been recognized as an effective, reproducible, and cost-effective technique for the management of idiopathic clubfoot worldwide, and 113 of 193 United Nation member states had adopted the Ponseti method as of 2014^8^.

The classification of the severity of deformity is important for pretreatment evaluation and monitoring treatment progress. Currently, the Dimeglio and Pirani scoring systems have become the most universally adopted classification systems, and have high intra-observer and inter-observer reliability, clinical relevance and can easily be used in clinical practice^9–12^. One of the questions most frequently asked by parents is how many casts will be needed before correcting the deformity, and accurately informing the parents improves patient treatment compliance. Several studies have investigated the correlation between initial Pirani score or Dimeglio score with the numbers of casts needed to acquire full correction of the deformity, but there is no consensus conclusion that has been drawn^11,13–15^. To our knowledge, there is no study that has investigated the correlation between initial Pirani score or Dimeglio score and the numbers of casts needed at different severities of the deformity.

The purposes of this study were two-fold: (1) to evaluate the interobserver reliability of initial Pirani score and Dimeglio score at different severities of the deformity, and (2) to evaluate the correlation between the initial Pirani scores or Dimeglio scores with the number of casts needed at different severities of the deformity.

## Results

### Spread of data points

**Figure 1:**
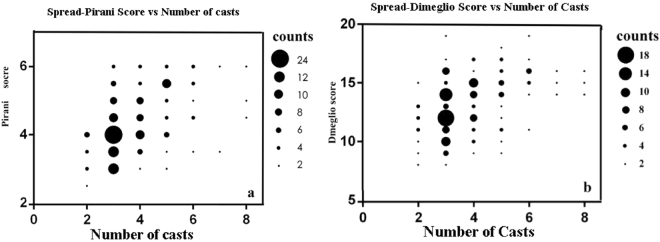
Spread of data points with the Pirani score (**a**) or Dimeglio score (**b**) systems and correlations with number of casts required to achieve deformity correction.

The spread of data points comparing initial Pirani scores or Dimeglio scores by the number of casts required to achieve deformity correction is shown in Fig. 1.

### The intraclass correlation coefficient (ICC) between initial Pirani and Dimeglio scores of feet with different severities of deformity

The reliability of initial Dimeglio scores or Pirani scores on the whole were substantial (ICC = 0.81 and 0.79), but with different numbers of casts, the ICCs were different. For feet with a cast number ranging from 3 to 6, the strength of agreement of the ICCs was substantial as well, with ICCs of 0.86, 0.82, 0.88, and 0.73 for initial Pirani score, respectively, and 0.81, 0.81, 0.82, and 0.71 for initial Dimeglio scores, respectively. However, for the feet with 2 or 7 and 8 casts, the reliability was poor or moderate with an ICC equal to 0.37 and 0.40 for initial Pirani score or 0.37 and 0.40 for initial Dimeglio score, respectively (Table 1).

**Table 1:** The intraclass correlation coefficients for the Pirani and Dimeglio scoring systems of the feet with different severities of deformity.

No. of casts	No. of cases	Pirani			Dimeglio		
		Rater 1	Rater 2	ICC	Rater 1	Rater 2	ICC
Total	173	4.428 ± 0.924	4.428 ± 1.5234	0.81	13.19 ± 2.388	12.65 ± 2.876	0.79
2	13	3.538 ± 0.5189	3.321 ± 0.5283	0.37	11.54 ± 1.854	10.96 ± 2.257	0.33
3	72	4.111 ± 0.8102	4.135 ± 0.8456	0.86	12.49 ± 2.301	13.56 ± 2.301	0.81
4	40	4.525 ± 0.8317	4.587 ± 0.8572	0.82	13.25 ± 2.085	13.41 ± 1.823	0.81
5	27	4.926 ± 0.8401	5.011 ± 0.8301	0.88	14.07 ± 2.433	13.89 ± 2.216	0.86
6	14	5.214 ± 0.7523	5.324 ± 0.7712	0.73	15.57 ± 1.785	15.42 ± 2.013	0.71
7 and 8	7	5.286 ± 0.9940	5.816 ± 1.1240	0.40	15.00 ± 0.816	15.39 ± 1.356	0.40

### The relation between initial Pirani and Dimeglio scores of different severities

The standardized severity of the deformity evaluated by the initial Pirani scoring system (0.738 ± 0.154) was more severe than that evaluated by the initial Dimeglio scoring system (0.660 ± 0.119), and the difference was statistically significant (*P* = 0.000) (Table 2).

**Table 2:** The standardized severity of the deformity evaluated by the Pirani and Dimeglio scoring systems.

No. of casts	No. of cases	Initial Pirani score/6	Initial Dimeglio score/20	*P* Value
Total	173	0.738 ± 0.154	0.660 ± 0.119	0.000

The initial Pirani and Dimeglio scores on the whole were highly correlated (*r* = 0.792, *P* = 0.000), but at different numbers of casts, the correlations were different. When the cast number ranged from 3 to 6, the reliability was high as well, with *rs* of 0.775, 0.758, 0.793, and 0.646, and *P* = 0.000, 0.000, 0.000, 0.012, respectively. However, when the number of casts was 2 or 7 and 8, the reliability was moderate or the correlation was high, *rs* of 0.453 or 0.719, respectively, but the *P*-values were 0.120 and 0.069, respectively, which indicated that there was no correlation between the initial Pirani and Dimeglio scores (Table 3).

**Table 3:** The relation between Pirani and Dimeglio scores of different severities.

No. of casts	No. of cases	Pirani	Dimeglio	*r*	*P* Value
Total	173	4.428 ± 0.924	13.19 ± 2.388	0.792	0.000
2	13	3.538 ± 0.5189	11.54 ± 1.854	0.453	0.120
3	72	4.111 ± 0.8102	12.49 ± 2.301	0.775	0.000
4	40	4.525 ± 0.8317	13.25 ± 2.085	0.758	0.000
5	27	4.926 ± 0.8401	14.07 ± 2.433	0.793	0.000
6	14	5.214 ± 0.7523	15.57 ± 1.785	0.646	0.012
7 and 8	7	5.286 ± 0.9940	15.00 ± 0.816	0.719	0.069

### The correlation of initial Pirani and Dimeglio score with the numbers of casts needed to achieve deformity correction

The *r* for the initial Pirani score and the number of casts was 0.495 (*P* = 0.000) and 0.426 (*P* = 0.000) for the initial Dimeglio score. For the classification of the initial Dimeglio system, only severe feet had a moderate correlation, with an *r* 0.390 (P = 0.000). For the moderate and very severe feet, there was no correlation with the variables (*r* = 0.280, 0.030, and *P* = 0.157, 0.873, respectively) (Table 4).

**Table 4:** The correlation between initial Pirani and Dimeglio scores with the numbers of casts.

Classification		Cases	Score	No of casts	*r*	*P*
Pirani	Total	173	4.428 ± 0.924	3.90 ± 1.312(2–8)	0.495	0.000
Dimeglio						
	Total	173	13.19 ± 2.388	3.90 ± 1.312(2–8)	0.426	0.000
	Moderate	27	9.52 ± 0.64	3.3 ± 0.82(3–8)	0.280	0.157
	Severe	115	14.15 ± 1.43	3.82 ± 1.29(2–8)	0.390	0.000
	Very severe	31	16.5 ± 0.85	4.7 ± 1.4(2–8)	−0.030	0.873

### Binary regression of the number of casts on initial Pirani scores or Dimeglio scores

The results of the curve estimation procedure showed that although all the r^2^ values were statistically significant (*P* = 0.000) for all curve estimation regression models, the largest r^2^ of 0.261 was found for Quadratic and Cubic models for initial Pirani scores, and the biggest r^2^ of 0.192 was found for the Cubic model for initial Dimeglio scores (Table 5).

**Table 5:** The results of 11 different curve estimation regression models used to regress the number of corrective casts on initial Pirani or Dimeglio score.

Curve estimation regression model	Initial Pirani scores vs numbers of casts		Initial Dimeglio scores vs numbers of casts	
	r^2^	*P* Value	r^2^	*P* Value
Linear	0.245	0.000	0.181	0.000
Inverse	0.259	0.000	0.184	0.000
Logarithmic	0.25	0.000	0.168	0.000
Quadratic	0.261	0.000	0.187	0.000
Cubic	0.261	0.000	0.192	0.000
Compound	0.235	0.000	0.174	0.000
Power	0.253	0.000	0.177	0.000
S-cure	0.248	0.000	0.163	0.000
Growth	0.235	0.000	0.174	0.000
Exponential	0.235	0.000	0.174	0.000
Logistic	0.235	0.000	0.174	0.000

## Discussion

Current management of clubfoot has moved towards conservative treatment using the Ponseti method, which has become the gold standard for the treatment of clubfoot. In our institution, clubfoot treatment was managed by a single surgeon (Z.L.) with rigid adherence to the Ponseti method such that treatment technique was consistent and the data were comparable^16^.

It is essential to develop a standardized, clinically relevant, easy to use and widely accepted clubfoot classification system to accurately evaluate the severity of the clubfoot deformity that can be used to monitor and guide treatment and to predict and identify early relapse^17^. The Pirani and Dimeglio classifications are the two most widely utilized scoring systems for clubfoot, and their intra-observer and inter-observer reliability has been shown to be good by their developers at their respective institutions^18,19^. A recent study showed that even the reliability test was carried out by five different orthopaedic surgeons, except for emptiness of the heel (EH), the total score and component clinical signs of the Pirani scoring system had substantial reliability with ICCs and Kappa values of posterior crease (PC), EH, rigidity of equinus (RE), hind foot score (HFS), medial crease (MC), curvature of lateral border (CLB), lateral head of talus (LHT), midfoot score (MFS), and total score (TS) were 0.4022, 0.3255, 0.6546, 0.6221, 0.4294, 0.5322, 0.534, 0.6407, and 0.7004 and 0.46, 0.39, 0.68, 0.66, 0.43, 0.56, 0.53, 0.68, and 0.71, respectively^10^. With a three-point classification of severity of each clinical sign, the Pirani scoring system may have little room for error between assessors. Therefore, Harvey *et al*. added a certainty measure score (0 or 1) to each sign to make a five-point severity scoring system, but there was no significant difference in reliability between the three-point and five-point severity scales^9,20^. Just by assessing the clubfoot from photographs, the inter-rater reliability of Pirani scores between 25 physiotherapists was fairly good regardless of their clinical experience^20^. Shaheen *et al*. also found that there was no significant difference in the inter-rater reliability between novice and experienced physiotherapists^21^. In one study, 2 senior staff paediatric orthopaedists independently and separately evaluated 280 children (411 feet) using the Dimeglio and Pirani scoring systems, and the inter-observer correlation coefficient for Dimeglio scores and Pirani scores were 0.85 and 0.89, respectively^12^. Our study produced the same results. The reliability of the initial Dimeglio score and Pirani score on the whole was substantial (ICC = 0.81 and 0.79, respectively). However, when evaluating different numbers of casts, the ICCs were different. When the cast number ranged from 3 to 6, the reliability was substantial, but when the number of casts was 2 or 7 and 8, the reliability was poor or moderate. Therefore, when the deformity was mild or very severe, the reliability of the initial Pirani and Dimeglio systems was poor. Under these conditions, the reliabilities could not objectively reflect the deformity of the clubfoot.

Our study showed that the standard initial severity of the deformity evaluated by Pirani scoring was more severe than that evaluated by Dimeglio scoring. To our knowledge, no study has reported this finding. We considered that it may be because the Dimeglio scoring system, which has a greater number of variables, may have some bias, and it may also be because the two scoring systems are different in nature. The Dimeglio scoring system evaluated reducibility, but the Pirani scoring system evaluated the morphologic aspects of the clubfoot.

Although both the Dimeglio score and the Pirani score have excellent inter-observer and intra-observer coefficients, their clinical utilities are different and complementary. The Dimeglio scoring system is based on the correction obtained after applying a gentle reduction of force on the deformed foot, and the presence of 4 severe signs. The Pirani scoring system is based on the physical appearance of the foot. To our knowledge, there was no research that had investigated the correlation between initial Dimeglio score and the Pirani score. We found that the two scoring systems were highly correlated with each other on the whole (*r* = 0.792, *P* = 0.000), but when the cast number was 2 or 7 and 8, there was no correlation between the two scoring systems (*r* = 0.453, *P* = 0.120 or *r* = 0.719, *P* = 0.069), and the inter-observer reliability was also poor (ICC = 0.37, 0.40 or 0.33, 0.40). This finding may have arisen because both scoring systems are subjective in nature and based solely on physical examination. For the clubfoot with moderate deformity, the two scoring systems had excellent inter-observer reliability, and the correlation between the two scoring systems was good. However, for a clubfoot with slight or very severe deformity, there may be some bias that caused poor inter-observer reliability. Moreover, the ICC of the initial Dimeglio scoring system was smaller than the Pirani scoring system, a result that is consistent with the results of a previous study^12^.

Despite their excellent intraobserver and interobserver reliabilities, no consensus has been reached on the correlation between initial Pirani or Dimeglio score and the number of casts required for deformity correction. P. J. Dyer found that the initial Pirani score had a strong link (*r* = 0.72, *P* = 0.0005) to the number of casts, and the correlation remained very high (0.66 and 0.79) when the patients were subdivided into tenotomy and non-tenotomy group, respectively^14^. In contrast, a study of 123 patients found the ICC for the initial total Pirani and Dimeglio scores and number of casts were 0.33 and 0.34, respectively^15^. Another study showed low (*r* = 0.21) or no (*r* = 0.12) correlation between initial Dimeglio or Pirani score, respectively, and the number of casts, as did the equinus (*r* = 0.21) and adduction (*r* = 0.17) individual components of the Dimeglio score, and posterior crease (*r* = 0.09) or adduction (*r* = 0.17) for the individual components of the Pirani score^11^. A recent study of 115 children (196 clubfeet) showed that there was no statistically significant difference in initial Pirani and Dimeglio scores between a recurrence and a non-recurrence group (P = 0.875, 0.624, respectively), but there was a statistically significant difference in the number of casts between the two groups (*P* < 0.001). What’s was interesting was that the initial grade of the Dimeglio score (I–IV) in the recurrent group (3.24 ± 1.41) was lower than the non-recurrent group (3.29 ± 1.04), but the number of casts in the recurrent group (5.58 ± 2.25,) was obviously higher than the non-recurrent group (4.05 ± 2.06) (*P* < 0.001)^22^. Therefore, with respect to recurrence, the correlation between the initial score of the two scoring systems and the number of casts remains unclear. In our study, we did not take the casts between percutaneous Achilles tenotomy (PAT) and the application of foot abduction orthosis (FAO) into account, and we concluded that the surgery can cause some bias. There were moderate correlations (*r* = 0.495, *P* = 0.000 or *r* = 0.426, *P* = 0.000) between initial total Pirani or Dimeglio scores and number of casts.

Anil Agarwal proposed an equation:

1 $${\rm{Number}}\,{\rm{of}}\,{\rm{casts}}=4.1+0.6\ast {\rm{initial}}\,{\rm{Pirani}}\,{\rm{score}}$$

(r^2^ = 0.05; multiple *r* = 0.24; *P* < 0.001), and the r^2^ of the equation was very small (0.05), which meant the goodness of fit of the model was very poor^13^. We found that the Quadratic and Cubic models may be perfect curve estimation regression models with high goodness of fit. The largest r^2^ were 0.261 and 0.192 for the Pirani and Dimeglio scores, respectively. This finding meant that there was a Quadratic or Cubic relation between initial Pirani or Dimeglio scores and the number of casts, and a larger number of casts was needed when the initial Pirani or Dimeglio scores were large.

There were several limitations in this study. First, the subjective nature of the two scoring systems may cause some bias. Second, we did not take component clinical signs of Pirani scoring system as the object of the study. Therefore, their relations to the total scores on the two scoring systems are unknown. We concluded that the score for the midfoot can more objectively reflect the effect of the cast on deformity correction because part of the deformity of the hindfoot was corrected by PAT. Third, we did not investigate the intra-observer reliability of initial Pirani and Dimeglio scoring system.

In conclusion, our research confirmed that when the deformity of the clubfoot was mild and very severe, the initial Pirani or Dimeglio scoring systems had poor inter-observer reliability, and under these conditions, there was no correlation between the two systems. When the severe deformity was moderate and very severe, the initial Dimeglio score or Pirani score had no correlation with the number of casts, and there was a Quadratic or Cubic relation between initial Pirani or Dimeglio scores and the number of casts. A more objective classification system may be needed.

## Methods

### Patients

A retrospective analysis of prospectively collected research data was carried out. The study was approved by the Xinhua Hospital Ethics Committee, and the informed consent from the patients was waived. All eligible patients had idiopathic clubfoot. Those with atypical or complex clubfeet, clubfeet with any treatment experience in other centres, a cast number less than two (for which the feet are regarded as postural clubfoot), or the interval to first cast over 3 months were excluded. For a patient who required PAT, the number of casts was equal to the time needing casts until PAT. For a patient who did not require PAT, the number of casts equalled the times needing a cast before Foot Abduction Orthosis (FAO).

Eventually, a total of 173 patients with 250 idiopathic clubfeet consecutively treated from January 2009 to December 2012 were recruited in our institution. For bilateral patients, we randomly selected either one or the one foot with a higher score or more casts needed if the severity of both feet was not the same as the object of the research^23^. Of the 173 patients, 42 (24.3%) were male, and 131 (75.7%) were female; 150 (86.7%) required PAT, and 23 (13.3%) did not require it. There were 77 (44.5%) patients with bilateral clubfeet and 96 (55.5%) with unilateral clubfoot. The right foot was involved in 37 (38.5%) of 96 cases and the left in 59 (61.5%) of 96 cases. The mean interval to first cast was 27.97 ± 19.73 (range 2–90) days. All of the patients were followed up for 35.2 (range, 26–58) months.

### Treatment method

In our institution, the patients were treated by a single orthopaedic surgeon (L.Z.) with rigid dedication to the Ponseti method for isolated clubfoot treatment. The method included several visits for manipulation and sequential plaster casting and then was followed by selective use of PAT lengthening procedures. When the deformity has been fully corrected, a long period of bracing and periodic follow-up was followed until the child was 4 years old. If relapse occurred, the patient was treated with a repeated procedure plus optional PAT or tibialis anterior tendon transfer surgery if necessary^24^.

### The Pirani and Dimeglio scoring systems

**Figure 2:**
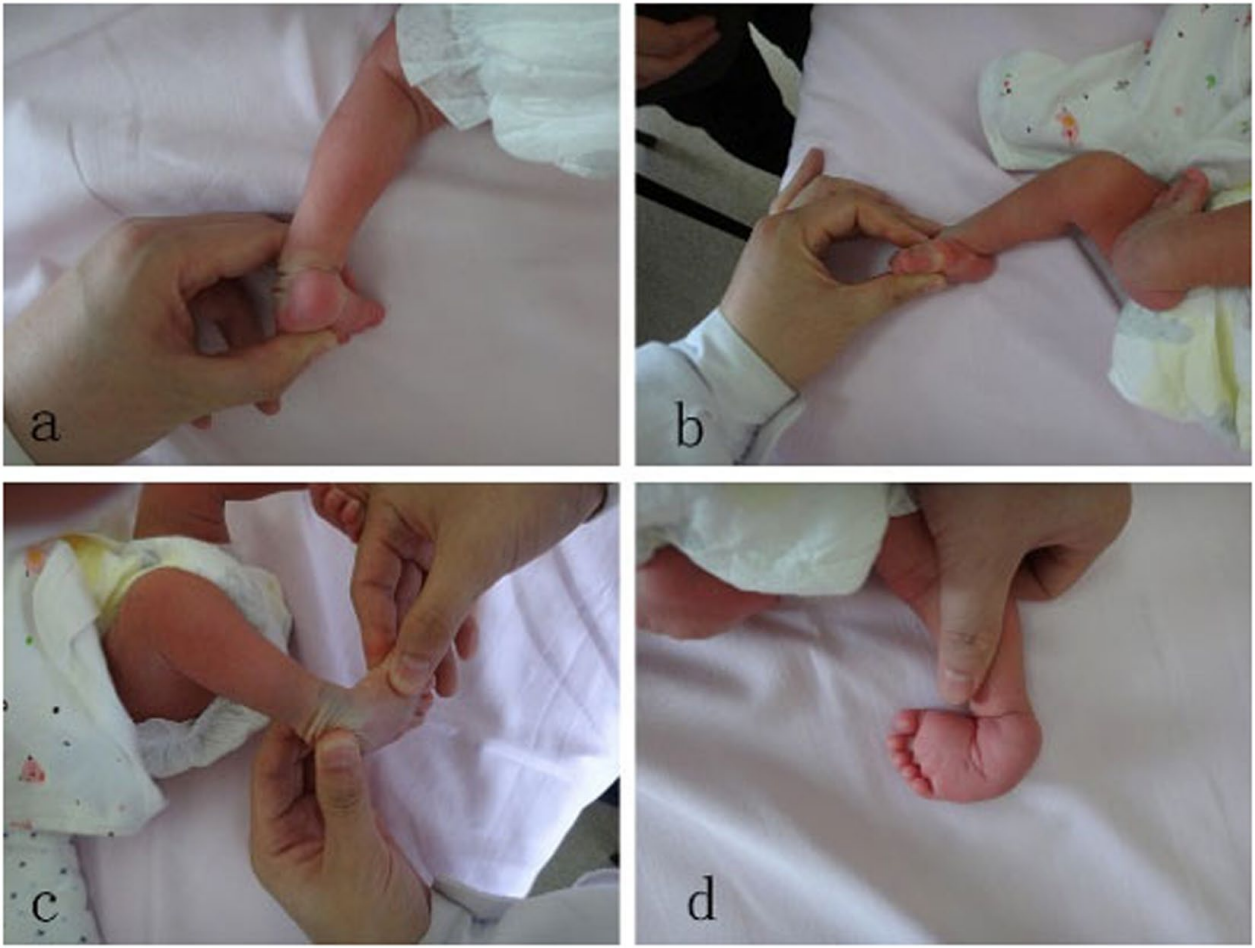
Pirai Score evaluation. **a**) Posterior heel crease = 0.5, **b**) Equinus = 1, **c**) Lateral talus head = 1, **d**) Medial crease = 1, Curvature of lateral border = 1.

**Figure 3:**
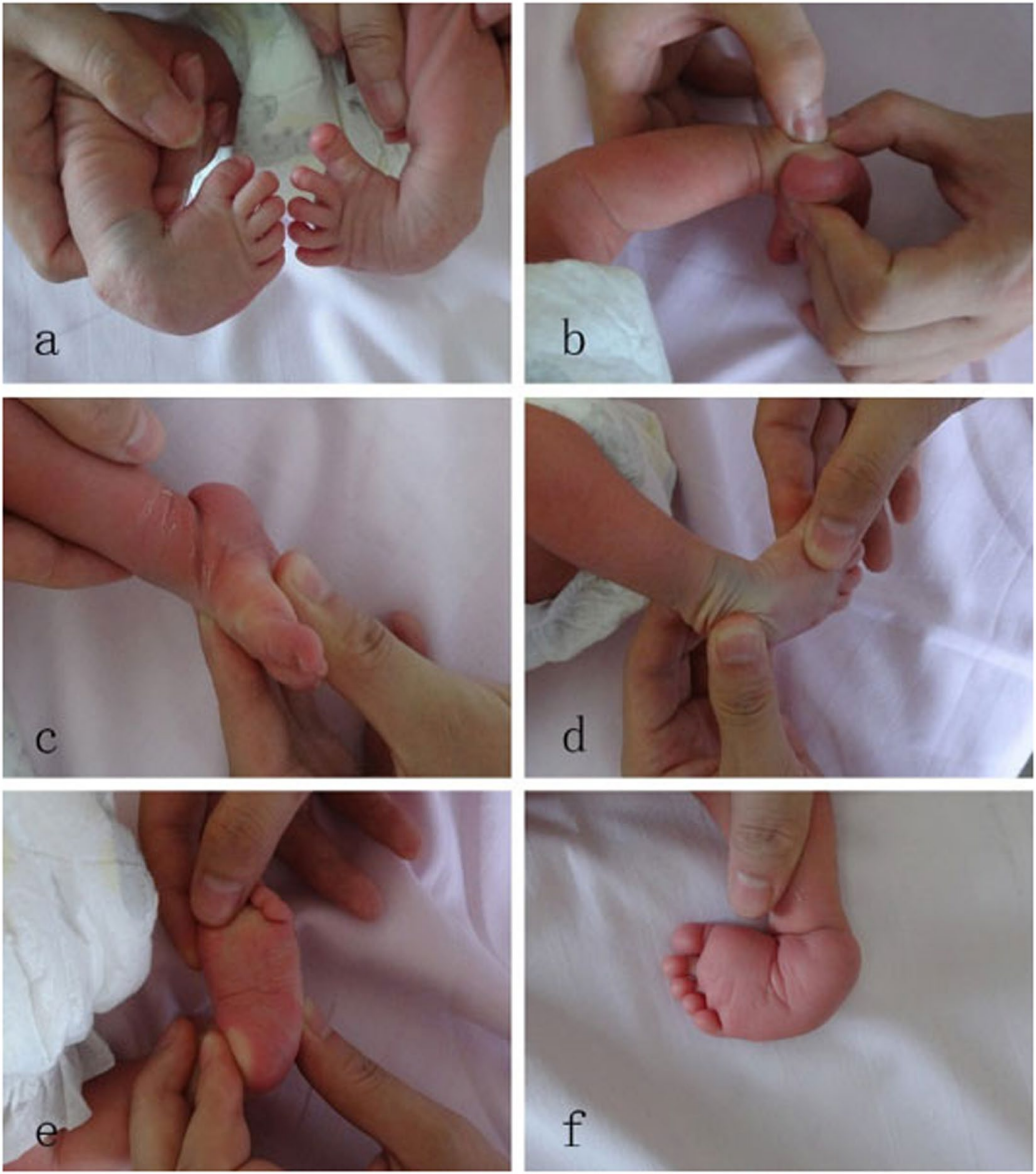
Dimeglio Score evaluation. **a**) Bilateral clubfoot, b) Varus = 3, **c**) Equinus = 4, **d**) Derotation of CFF = 2, **e**) Adducuts = 2, **f**) Cavus = 1, medial crease = 1.

The patients were reviewed by the two authors (F.H. and L.Y. B.) using the Pirani or Dimeglio scoring systems before initial cast correction. The Pirani classification has 6 clinical signs of deformity, and both midfoot score and hindfoot score have 3 signs. Each sign is scored as 0, 0.5 or 1 corresponding to no, moderate or severe abnormality, respectively. Therefore, there is a total score of between 0 and 6 for a foot^19^ (Fig. 2). The Dimeglio scoring system includes the visual estimation of the equinus, hind foot varus, midfoot rotation and forefoot adduction without forcing the foot, and each feature is given 0 to 4 points according to reducibility (90–45° = 4; 45–20° = 3; 20–0° = 2; 0°–20° = 1; less than 20° = 0) on the relative plane, and pejorative elements (posterior crease, medial crease, cavus and muscular abnormality, MA) were each scored as 1 if present and 0 if absent. The total scale ranged from 0 to 20, with a score of 0 for a normal foot, ≤5 a benign deformity foot, 6–10 a moderate deformity foot, 11–15 a severe deformity foot and 16–20 a very severe deformity foot^18^ (Fig. 3). The severity of the deformity for each foot was standardized by initial Pirani score divided by 6 and initial Dimeglio score divided by 20.

### Statistical analysis

The quantitative variables are expressed as the mean ± standard deviation. The intra-class correlation coefficients (ICCs; 2-way random effects model, single-measure reliability) were calculated for initial Pirani and Dimeglio scores acquired by the two reviewers. A score of 0.81 to 1 were rated as very good, 0.61 to 0.8 as good, 0.41 to 0.6 as moderate, and less than 0.4 as poor reliability^10^. The difference in standardized deformity between the Pirani score and Dimeglio scores was calculated by paired sample t-test. The relationship between the initial Pirani score and Dimeglio score was established using the Pearson correlation coefficient, and their relation to the number of Ponseti casts for each foot was established using the Spearman rank correlation coefficient. The correlation coefficients ranging from 0 to 0.2 are regarded as having no correlation, 0.20–0.40 was a low correlation, 0.4–0.6 was a moderate correlation, 0.6–0.8 was a marked correlation and 0.8–1.00 was a high correlation^25^. Eleven curve estimation regression models (Linear, Logarithmic, Inverse, Quadratic, Cubic, Compound, Power, S-curve, Growth, Exponential, and Logistic) were applied to find the best mathematical equation to calculate the number of corrective casts with initial Pirani and Dimeglio score, and the corresponding coefficient of determination (r^2^) was calculated.
